# A novel pathway of levodopa metabolism by commensal *Bifidobacteria*

**DOI:** 10.1038/s41598-023-45953-z

**Published:** 2023-11-06

**Authors:** M. S. Cirstea, A. Creus-Cuadros, C. Lo, A. C. Yu, A. Serapio-Palacios, S. Neilson, S. Appel-Cresswell, B. B. Finlay

**Affiliations:** 1 Department of Microbiology and Immunology, University of British Columbia (UBC), Vancouver, BC Canada; 2 Michael Smith Laboratories, University of British Columbia, 2185 East Mall, Vancouver, BC V6T 1Z4 Canada; 3 Pacific Parkinson’s Research Centre, UBC, Vancouver, BC Canada; 4 Division of Neurology, Faculty of Medicine, UBC, Vancouver, BC Canada; 5 Department of Biochemistry and Molecular Biology, UBC, Vancouver, BC Canada

**Keywords:** Microbiome, Parkinson's disease

## Abstract

The gold-standard treatment for Parkinson’s disease is levodopa (L-DOPA), which is taken orally and absorbed intestinally. L-DOPA must reach the brain intact to exert its clinical effect; peripheral metabolism by host and microbial enzymes is a clinical management issue. The gut microbiota is altered in PD, with one consistent and unexplained observation being an increase in *Bifidobacterium* abundance among patients. Recently, certain *Bifidobacterium* species were shown to have the ability to metabolize L-tyrosine, an L-DOPA structural analog. Using both clinical cohort data and in vitro experimentation, we investigated the potential for commensal *Bifidobacteria* to metabolize this drug. In PD patients, *Bifidobacterium* abundance was positively correlated with L-DOPA dose and negatively with serum tyrosine concentration. In vitro experiments revealed that certain species, including *B. bifidum*, *B. breve*, and *B. longum*, were able to metabolize this drug via deamination followed by reduction to the compound 3,4-dihydroxyphenyl lactic acid (DHPLA) using existing tyrosine-metabolising genes*.* DHPLA appears to be a waste product generated during regeneration of NAD +. This metabolism occurs at low levels in rich medium, but is significantly upregulated in nutrient-limited minimal medium. Discovery of this novel metabolism of L-DOPA to DHPLA by a common commensal may help inform medication management in PD.

## Introduction

The hallmark pathology of Parkinson’s disease (PD) is the loss of dopamine-producing neurons in the substantia nigra, leading to a dopamine deficit in the striatum^1,2^. PD has no cure, with current treatments aimed at providing symptom relief. The gold standard among these treatments is levodopa (L-DOPA), which is prescribed to the vast majority of PD patients within the first few years of diagnosis^3^. This direct dopamine precursor is taken orally, absorbed in the small intestine, travels to the brain via the circulatory system, and is converted to dopamine inside neurons by an L-aromatic amino acid decarboxylase (AADC)^1,3^. L-DOPA is usually taken several times per day, with the goal of keeping its circulating concentration within a critical window of therapeutic efficacy. Concentrations below this effective window lead to bradykinesia (slowness of voluntary muscle movement) and rigidity, and above lead to dyskinesia (uncontrolled, involuntary movement)^4,5^.

Dopamine cannot cross the blood–brain barrier (BBB), therefore almost all PD patients taking L-DOPA simultaneously take an AADC inhibitor such as carbidopa to prevent peripheral L-DOPA conversion into dopamine^1,3^. L-DOPA can also be metabolized by enzymes such as catechol O-methyltransferase (COMT), thus COMT inhibitors such as entacapone are also frequently prescribed^1,3^. These inhibitors cannot cross the BBB; therefore, they preserve L-DOPA in the periphery without impacting its conversion to dopamine in the brain^3^. Preventing peripheral metabolism prolongs L-DOPA’s clinical effect, lowers the overall dose required, and reduces the side effects that can be caused by downstream metabolites: for instance, high levels of circulating dopamine can cause nausea, vomiting, and low blood pressure^3^.

Current inhibitors of L-DOPA metabolism target host enzymes. However, it has recently been recognized that certain bacteria also have the potential to metabolize L-DOPA in the gut^6,7^. To date, this research has focused on metabolism of L-DOPA to dopamine by microbial tyrosine decarboxylases (TDCs). The first paper to describe this phenomenon found relevant TDC enzymes in members of the Bacilli class, especially *Enterococcus* species^6^*.* These TDCs, which normally function to decarboxylate tyrosine to tyramine, were extremely efficient in the decarboxylation of L-DOPA to dopamine in vitro; and when rats were administered oral L-DOPA, the concentration that reached circulation was inversely proportional with TDC gene abundance in their guts^6^. Importantly, carbidopa did not inhibit these bacterial enzymes^6^. A second study from the same year made a similar discovery related to TDC enzymes in *Enterococcus*, and found that the bacterium *Eggerthella lenta* can sequentially dehydroxylate dopamine to tyramine^7^. The authors also showed that the tyrosine analog (S)-α-fluoromethyltyrosine can inhibit the bacterial TDC enzymes and prevent L-DOPA decarboxylation, demonstrating the first proof of principle that inhibition of bacterial metabolism may be another tool to preserve L-DOPA’s clinical efficacy.

**Figure 1:**
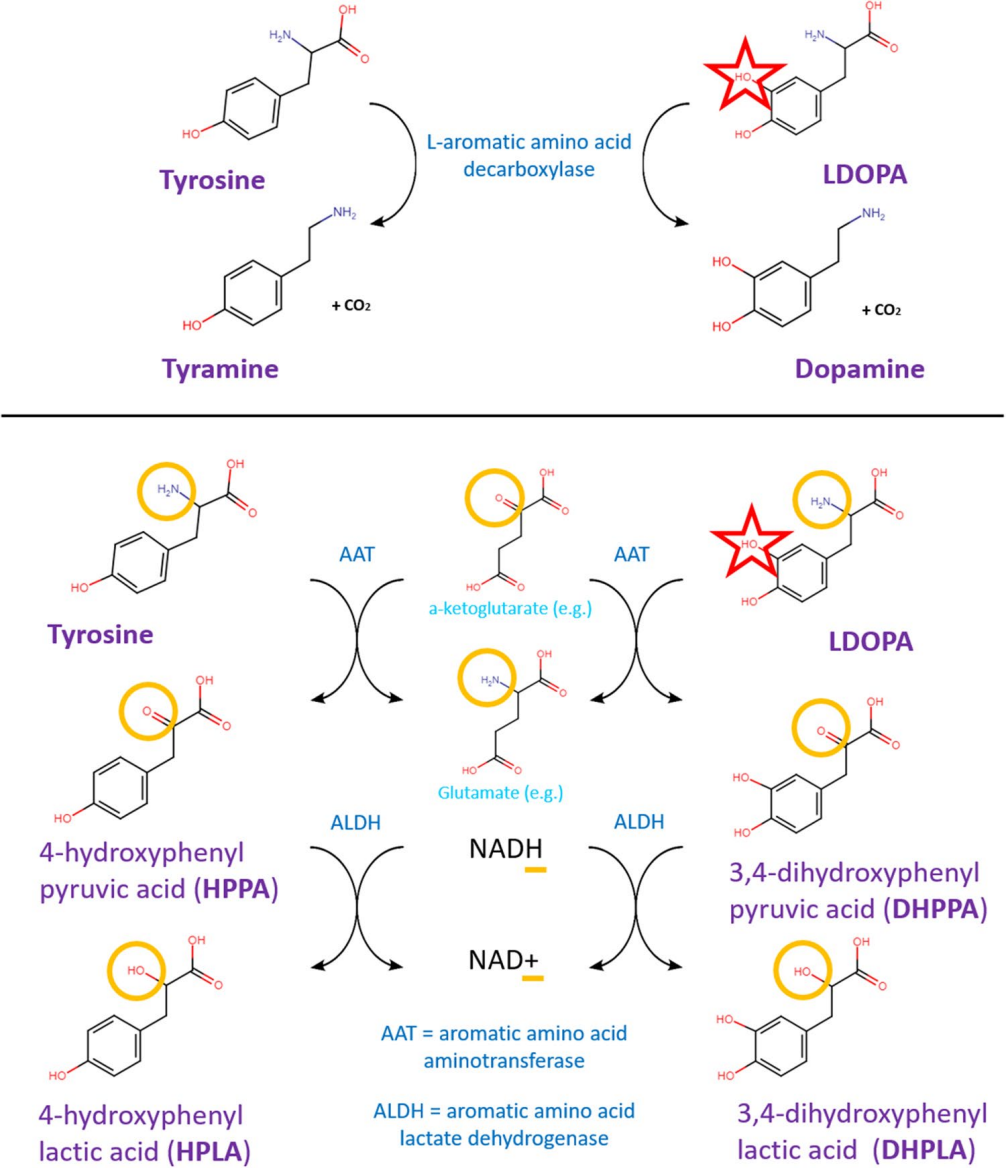
Proposed metabolism pathways. L-DOPA metabolism (right) and analogous pathways of tyrosine metabolism (left). The only difference between the two molecules is a hydroxy group on the unmetabolized aromatic ring, highlighted by a red star. TOP: Decarboxylation of tyrosine to tyramine (left) and of L-DOPA to dopamine (right), a pathway known to exist in *Enterococcus* spp. BOTTOM: Deamination and reduction of tyrosine to HPLA (left), a pathway recently shown to exist in certain *Bifidobacterium* spp; and the proposed analogous pathway of L-DOPA conversion to DHPLA (right).

Although decarboxylation has been the primary focus, other metabolic pathways may exist among the myriad enzymes encoded by the gut microbiota. Given the above TDC findings, and since tyrosine and L-DOPA differ in structure by only a single hydroxy group, existing tyrosine metabolism enzymes are the most likely sources of potential L-DOPA metabolism. Tyrosine can also be metabolized to 4-hydroxyphenyl lactic acid (HPLA) through a two-step reaction involving deamination by an aromatic amino acid aminotransferase (AAT) followed by reduction by an aromatic amino acid lactate dehydrogenase (ALDH). Lactate dehydrogenases are common among pathogens and environmental bacteria, but rarer among commensal gut microbes. However, it was recently found that certain *Bifidobacterium* species possess a previously unrecognized ALDH, and can produce lactic acids from aromatic amino acids including tyrosine^8^. This is intriguing as *Bifidobacterium* is one of the primary genera consistently seen at higher relative abundance in PD^9–11^, including in our own study^12^. Analogous to the ability of *Enterococcus* TDCs to convert L-DOPA to dopamine, we hypothesized that *Bifidobacteria* can use native tyrosine-metabolism enzymes to convert L-DOPA to its equivalent lactic acid, 3,4-dihydroxyphenyl lactic acid (DHPLA) (Fig. 1).

## Methods

### Participant recruitment and data collection

The participant cohort used in this study was recently described by our group^12^, including extensive methods on clinical data collection, fecal 16S microbiota analysis, and serum metabolomics analysis. Briefly, 197 PD patients and 103 controls were recruited from the Pacific Parkinson’s Research Centre, University of British Columbia, between January 2017 and September 2019. During a study visit, a wide range of demographic and clinical data were collected, including L-DOPA dose. Participants were sent home with a fecal collection kit (OMNIgeneGUT Kit, DNA Genotek) and instructed to provide a fecal sample and mail it back to the centre at the next available opportunity; this kit contains a buffer which halts microbial growth and provides a snapshot of the microbiota at the moment of collection. Gut microbiota composition was analyzed using 16S sequencing of the V4 region, as described^12^. Notably, participant exclusion criteria included recent antibiotic and probiotic use, therefore any *Bifidobacterium* detected in their microbiota was expected to be stably colonized and not a result of recent probiotic ingestion. A small subset of patients from this study provided a fresh fecal sample on site during their visit, which was collected into a 50 mL conical tube and immediately flash frozen in liquid nitrogen then stored in a − 70 °C freezer. Isolated colonies used in this study originated from these flash-frozen samples. For methodological consistency, these participants also provided a preserved sample (OMNIgene GUT Kit) for use in 16S analysis. During the clinical visit, a blood sample was collected and untargeted metabolomics were performed on serum samples from 75 PD patients and 50 controls^12^.

This study was approved by the UBC Clinical Research Ethics Board (CREB) and conformed to all relevant CREB guidelines. All participants provided written informed consent. The study was performed in accordance with the Declaration of Helsinki.

### Isolation of *Bifidobacterium* strains from PD fecal samples

On the day of isolation, the flash-frozen tube containing the freshly collected fecal sample was taken on ice into an anaerobic chamber, and a small amount of fecal material was added to a 2 mL Eppendorf tube containing 1 mL PBS with 0.1% cysteine. The sample was left on ice in the chamber for 30 min, then gently inverted until the sample appeared relatively homogenous, and larger particles were left to settle for 5 min. 100 µL was then plated onto de-gassed De Man, Rogosa and Sharpe plates with 0.1% cysteine (MRS + C) using glass bead spread plating. The plates were incubated at 37 °C in the anaerobic chamber.

The following day, 20–30 colonies per plate were lightly touched with a sterile pipet tip and streaked onto new labelled MRS + C plates, then the tip was touched into a strip tube containing a pre-made 25 µL PCR mix (Q5 Hot Start High-Fidelity 2X Master Mix, NEB M0494L) and 8F/926R 16S primers (F: AGAGTTTGATCCTGGCTCAG, R: CCGTCAATTCCTTTRAGTTT). The newly streaked plates were returned to the incubator, and the PCR was run with the following conditions: 98 °C for 5 min, followed by 35 cycles of 98 °C for 20 s, 55 °C for 15 s, 72 °C for 45 s, then 72 °C for 10 min and a 4 °C hold. The reactions were cleaned using GeneJET PCR Purification kits (ThermoFisher K0701) and sent for Sanger sequencing (GeneWiz) using the 16S-8F primer. The resulting 600–700 bp sequences were interrogated using NCBI BLAST. Bacteria of interest were re-streaked onto another new plate, re-sequenced to confirm their identity, then grown up in 5 mL of liquid MRS + C broth from which glycerol stocks were made. Isolated bacteria were named using the convention “*Genus species_*##” where the identity was determined by Sanger sequencing of the 16S gene and the “##” refers to the anonymized patient ID that they were derived from. The following strains were isolated: *B. pseudocatenulatum_03, B. longum_03, B. animalis_04, B. adolescentis_05, B. stercoris_07, B. longum_07, B. longum_08,* and *Enterococcus faecalis_01.* In addition, the following species were obtained from the German Collection of Microorganisms and Cell Cultures (DSM-Z): *B. bifidum* (DSM-20456), *B. breve* (DSM-20213), and two environmental *Lactobacilli*: *L. plantarum* (DSM-20174) and *L. fermentum* (DSM-20052). *B. adolescentis* (ATCC-15703) and *B. longum* subsp. *longum* (ATCC-15707) (existing Finlay lab stocks) were also used. All bacteria were routinely grown anaerobically at 37 °C using degassed MRS + C medium.

### Screening for tyrosine decarboxylase (TDC) activity

Bacterial isolates were screened for TDC activity using decarboxylation medium agar plates^13^ containing basal nutrient sources, with or without 1% tyrosine. Medium composition is listed in Supplementary Table 1. The pH indicator bromocresol purple changes colour from yellow to purple if the more acidic amino acid (tyrosine) is converted to the more alkaline biogenic amine (tyramine). Bacteria were plated and grown anaerobically for 48 h at 37 °C. A colour change from yellow to purple on tyrosine plates, accompanied by no colour change on non-tyrosine control plates, indicated TDC activity.

### Screening for tyrosine metabolism to HPLA

Bacteria were screened for their ability to convert tyrosine to 4-hydroxyphenyl lactic acid (HPLA) using mass spectrometry (MS). Bacteria were plated on MRS + C plates and individual colonies were transferred to 5 mL MRS + C broth overnight. Cultures were diluted to OD_600_ = 0.01 in fresh MRS + C medium containing 0.01% pyridoxal 5'-phosphate (P5P), in a final volume of 1.2 mL in 2 mL snap-cap Eppendorf tubes. Tubes were anaerobically incubated for 48 h in a 37 °C shaking incubator (Eppendorf ThermoMixer C). Cultures were then spun at 16,000 × g at 4 °C for 10 min, and the clarified supernatant was aliquoted to new tubes and frozen at − 70 °C.

Metabolite extraction was performed as follows: samples were thawed on ice and spun at 16,000 × g at 4 °C for 5 min. 80 µL of supernatant was added to tubes containing 320 µL acetonitrile + 0.1% formic acid. Samples were vortexed for 10 s then incubated at − 20 °C for 10 min, during which two distinct phases formed: a bottom turbid phase and a top translucent solvent phase. Samples were spun at 16,000 × g at 4 °C for 10 min to further separate the phases, and then 50 µL of the top phase was added to a tube containing 50 µL of H2O + 0.1% formic acid and 200 ng/µL deuterated L-DOPA-d_3_ (MS internal standard, Cayman Chemicals 22089). The final sample was therefore a 1:10 dilution of the original, in 50% acetonitrile: 50% H2O + 0.1% formic acid, with 100 ng/µL internal standard. Metabolite extractions were prepared immediately prior to MS runs.

### Screening for L-DOPA metabolites

Experiments were performed as above, with the addition of the relevant metabolites (L-DOPA, 3,4-dihydroxyphenyl pyruvic acid (DHPPA), or 3,4-dihydroxyphenyl lactic acid (DHPLA)) at a final concentration of 1 mM, as well as 0.25% ascorbic acid to prevent L-DOPA oxidation. Compounds used for experiments and for MS analytical standards were sourced as follows: L-DOPA (Toronto Research Chemicals D533751), dopamine (Cayman Chemicals 21992), DHPLA (TargetMol T3227), DHPPA (AChemBlock M15562), HPLA (Sigma H3253).

### Mass spectrometry parameters

An LC–MS/MS system equipped with an Agilent Technologies 1200 high-performance liquid chromatography (HPLC) instrument, an Agilent 6460 triple quadrupole (QQQ) mass spectrometer, and a reversed-phase Nucleosil 100 C18 5 µm particle size column (Supelco) was used for MS runs. MS was conducted in positive and negative ion mode with an electrospray ionization voltage of 3500 V with 50 psi nebulizer gas at a temperature of 350 °C. Sample injection volume was 5 µL. LC separation was performed using mobile phases A (0.1% formic acid, 3% acetonitrile, 97% H_2_O) and B (0.1% formic acid, 90% acetonitrile, 10% H_2_O), at a flow rate of 400 µL/min. The gradient program began with 15% B, increased linearly to 70% B over 7 min, was held for 1 min, then returned to 15% B over 1 min and was held for 3 min. A collision energy of 10 V was used for multiple-reaction monitoring. Data were analyzed using Mass Hunter Qualitative Analysis B.06.00 software (Agilent Technologies). Positive/negative ion mode was selected according to the best ionization efficiency: dopamine, L-DOPA and L-DOPA-d_3_ were run in positive; and HPLA and DHPLA were run in negative mode. Metabolite identification and quantification were carried out based on the retention time and mass fragmentation patterns compared with analytical standards. Six-point calibration curves were prepared, combining L-DOPA, dopamine, DHPLA, and HPLA together at final concentrations from 4000 to 10 ppb, all with 100 ppb of L-DOPA-d_3_ as an internal standard. The R^2^ for linearity was > 0.99 for all compounds. Metabolite concentrations were calculated by interpolating against calibration curves of individual compounds. LC–MS/MS instrument settings are available in Supplementary Table 2.

### Enzymatic assays with cell-free extracts

Cell-free extracts (CFEs) for enzymatic assays were prepared based on an existing protocol^14^. Bacteria were grown for 16 h in 15 mL MRS + C broth, then spun for 15 min at 4800 × g at 4 °C. The supernatant was discarded and pellets were washed twice with 10 mL PBS using the same spin parameters. After the second wash, pellets were resuspended in 2 mL PBS and cells were lysed by sonication with a Fisher 550 Sonic Dismembrator on ice: [15 s on/15 s off] for 5 min total time at 20 kHz speed. Lysates were spun as above and the enzyme-containing supernatants were aliquoted and frozen at − 70 °C.

Two 96-well-plate-based enzymatic assays were performed using these CFEs, as described^14^. First, aromatic amino acid aminotransferase (AAT) activity was assayed by monitoring conversion of tyrosine/L-DOPA to HPPA/DHPPA, respectively, by transfer of an amino group onto α-ketoglutarate, which causes an increase in absorbance at A_305_. Second, aromatic amino acid lactate dehydrogenase (ALDH) activity was assayed by monitoring reduction of HPPA/DHPPA to HPLA/DHPLA, respectively, using NADH as a substrate and measuring the decrease in absorbance at A_340_ as NADH is converted to NAD +. For both assays, reactions without substrate and without CFEs were included as controls. Absorbance was measured in two minute intervals using a BioTek Synergy H1 plate reader.

### Development of a *Bifidobacterium* minimal media

A *Bifidobacterium* minimal medium (BMM) was developed based on existing literature^15^. This defined medium uses lactose as a sole carbon source and ammonium acetate as a sole nitrogen source. Importantly, it also contains pyruvate, which is oxidized by the bacteria to regenerate NAD + from NADH. Full medium composition is listed in Supplementary Table 3. This medium was prepared with and without 1% casamino acids (CAA) as many *Bifidobacterium* species are auxotrophs for certain amino acids. All *Bifidobacterium* strains were able to grow on BMM + CAA, but only *B. breve* grew without CAA, displaying no amino acid auxotrophy. As L-DOPA metabolism involves amino acid metabolism pathways, further work with this minimal medium focused on *B. breve* in order to exclude exogenous amino acids.

### Statistical analysis

The difference in *Bifidobacterium* relative abundance between PD patients and controls was assessed using *DESeq2*^16^ with multiple testing correction for all bacterial genera, as we previously described^12^. Demographic and clinical differences between patients with low versus high *Bifidobacterium* abundance were assessed using Mann–Whitney U tests for continuous variables and Fisher’s exact tests for categorical variables. Associations between *Bifidobacterium* abundance and serum metabolites were tested using Spearman correlation. Production of L-DOPA metabolites by mass spectrometry and enzymatic activity assays were assessed qualitatively. The difference in *B. breve* growth in various media conditions was assessed using a one-way ANOVA with Tukey’s multiple comparison test.

## Results

### *Bifidobacterium* abundance is elevated in PD patients, and is associated with higher L-DOPA dosage

**Figure 2:**
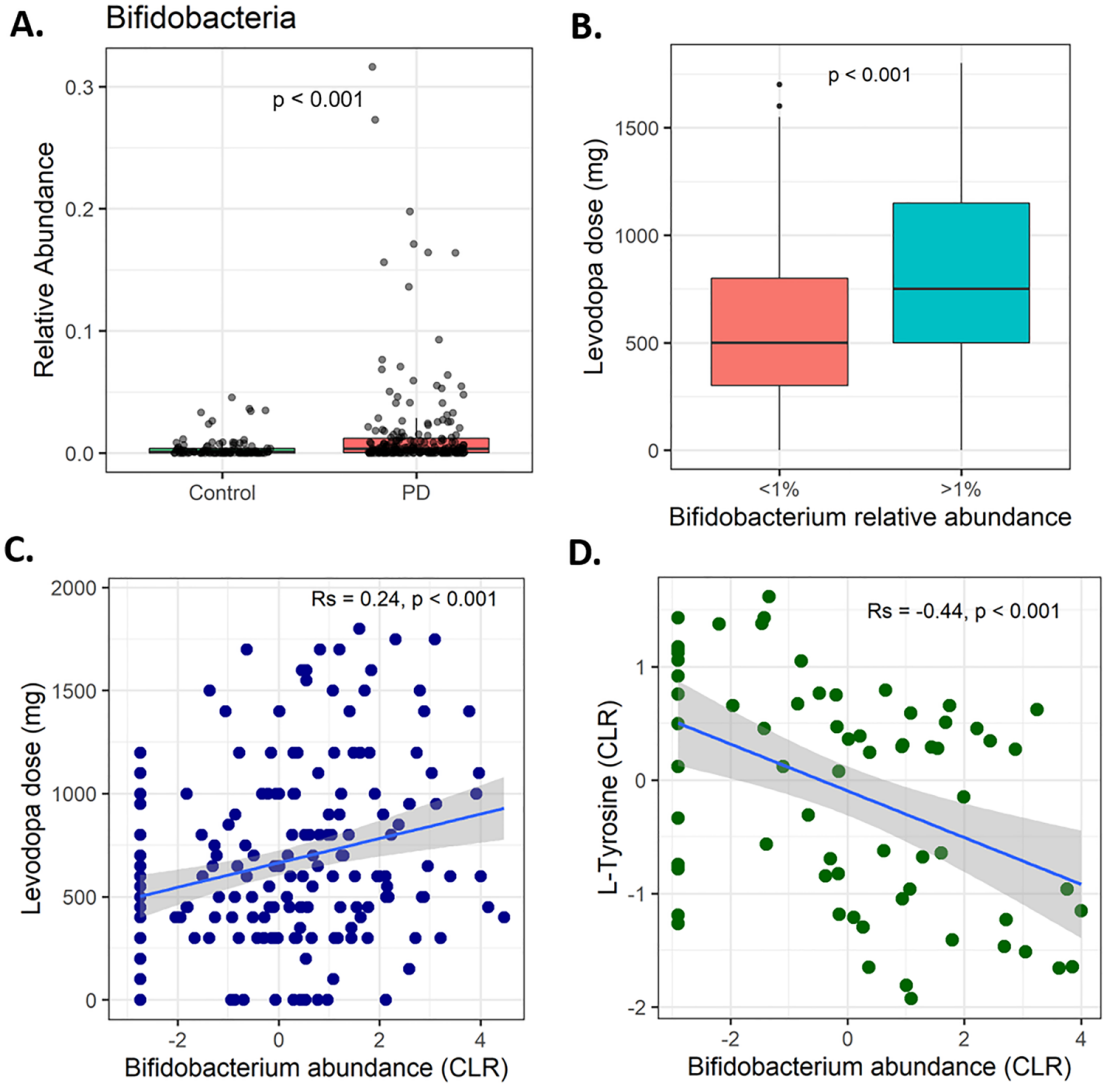
Human clinical data. (**A**) PD patients had a higher relative abundance of *Bifidobacterium* than controls. (**B**) PD patients with *Bifidobacterium* relative abundance > 1% had a significantly higher L-DOPA dose. (**C**) *Bifidobacterium* abundance (center-log ratio transformed) was positively correlated with L-DOPA dose in PD patients. (**D**) *Bifidobacterium* relative abundance was negatively correlated with serum tyrosine concentration.

In our cohort of 197 PD patients and 103 controls, we observed a significantly higher relative abundance of *Bifidobacterium* among PD patients (Fig. 2A), consistent with numerous published studies^9^. In order to investigate potential clinical differences between PD patients with lower versus higher levels of *Bifidobacterium,* we divided the patient cohort into two groups based on a nominal threshold of 1% *Bifidobacterium* relative abundance in their gut microbiota (Table 1). These groups were well matched on basic demographics (age, sex, BMI). Those with *Bifidobacterium* > 1% had a longer disease duration (8 vs 6 years) and slightly higher disease severity (MDS-UPDRS^17^) scores, especially MDS-UPDRS Part IV which queries motor fluctuations which are typically caused by reduced duration and reliability of anti-parkinsonian medication^18^.

**Table 1:** Demographics and clinical characteristics of PD patients with *Bifidobacterium* relative abundance in their gut microbiota below versus above 1%.

	*Bifidobacterium* abundance < 1%	*Bifidobacterium* abundance > 1%	P value
N =	141	56	
Basic demographics			
Female, N (%)	55 (39.0%)	20 (35.7%)	0.75
Age in years (median, IQR)	66 (59–71)	66 (59–71)	0.75
Body mass index (median, IQR)	26.2 (22.9–29.0)	25.6 (23.0–29.7)	0.96
Age of PD onset (median, IQR)	59 (54–66)	58 (52–63)	0.14
Disease duration (median, IQR)	6 (3–8)	8 (3.5–12)	**0.019**
Levodopa dose and other parkinsonian medication use			
Levodopa dose mg, mean (95% CI)	586 (519–655)	838 (721–956)	** < 0.001**
Levodopa equivalent dose^20^, mean (95% CI)	583 (521–646)	916 (784–1049)	** < 0.001**
Entacapone use, N (%)	2 (1.4%)	15 (26.8%)	** < 0.001**
Dopamine agonist use, N (%)	19 (13.5%)	14 (25%)	0.059
MOA-B inhibitor use, N (%)	18 (12.8%)	12 (21.4%)	0.13
Amantadine use, N (%)	6 (4.3%)	5 (8.9%)	0.30
Parkinson's severity scores			
MDS-UPDRS Part I score (median, IQR)	9 (5–13)	10 (7.5–14)	0.061
MDS-UPDRS Part II score (median, IQR)	8 (4.5–13.5)	11 (5.5–14)	0.22
MDS-UPDRS Part III score (median, IQR)	21 (16–29)	23 (15–30)	0.94
MDS-UPDRS Part IV score (median, IQR)	1 (0–3)	3 (1–5)	** < 0.001**
MDS-UPDRS total score (median, IQR)	42 (31.5–51)	48.5 (38.5–60.5)	**0.033**

Significant values are in bold.

The most striking difference between the groups was that patients with *Bifidobacterium* abundance greater than 1% had an average L-DOPA dose 252 mg (43.0%) higher than those with *Bifidobacterium* less than 1% (Mann–Whitney U test p < 0.001, Fig. 2B). There was also a positive correlation between numerical *Bifidobacterium* abundance and L-DOPA dose (R_s_ = 0.24, p < 0.001, Fig. 2C)^19^.

Patients with higher *Bifidobacterium* also had a significantly higher rate of entacapone use. Entacapone is a catecholamine-O-methyltransferase (COMT) inhibitor which prevents degradation of L-DOPA by the enzyme COMT and prolongs its half-life in the body^19^. When excluding patients taking entacapone, the L-DOPA dosage difference between patients with *Bifidobacterium* abundance below and above 1% remained significant (585 mg vs. 774 mg, 32.3% difference, Mann–Whitney U test p = 0.025).

To further investigate the association between *Bifidobacterium* and L-DOPA, a correlational analysis was performed using serum metabolomics data^12^, correlating all putative metabolite concentrations with *Bifidobacterium* relative abundance. Interestingly, the strongest correlation between any metabolite and *Bifidobacterium* abundance was annotated as L-tyrosine in the Human Metabolome Database^21^. *Bifidobacterium* and L-tyrosine concentration displayed a negative correlation (R_s_ =  − 0.44, p < 0.001, Fig. 2D). The positive correlation between *Bifidobacteria* and L-DOPA dose, coupled with the negative correlation between *Bifidobacteria* and serum tyrosine, is consistent with the idea that *Bifidobacteria* are able to metabolize tyrosine (leading to a negative correlation) and perhaps L-DOPA using analogous pathways (leading to a higher dosage requirement, and/or the higher doses creating a gut environment that confers some growth advantage to *Bifidobacterium*).

### *Bifidobacteria* do not decarboxylate L-DOPA to dopamine

Decarboxylation medium plates revealed a lack of tyrosine decarboxylation activity among all *Bifidobacterium* isolates screened, with *Enterococcus faecalis*, a bacterium with known tyrosine decarboxylase ability^6^, serving as a positive control (Supplementary Fig. 1). As the decarboxylation medium plates may not have had suitable sensitivity for detecting very low amounts of dopamine production, we further interrogated this via mass spectrometry, where our detection limit was 62.5 ng/mL. All *Bifidobacterium* strains, and *E. faecalis*, were incubated with 1 mM L-DOPA for 48 h and production of dopamine was quantified by LC–MS/MS. *E. faecalis* produced 1 mM dopamine, representing ~ 100% decarboxylation of L-DOPA, while dopamine was undetectable in all *Bifidobacterium* samples. Therefore, the ability of *Bifidobacteria* to decarboxylate L-DOPA to dopamine was ruled out.

### Tyrosine metabolism by *Bifidobacteria*

**Figure 3:**
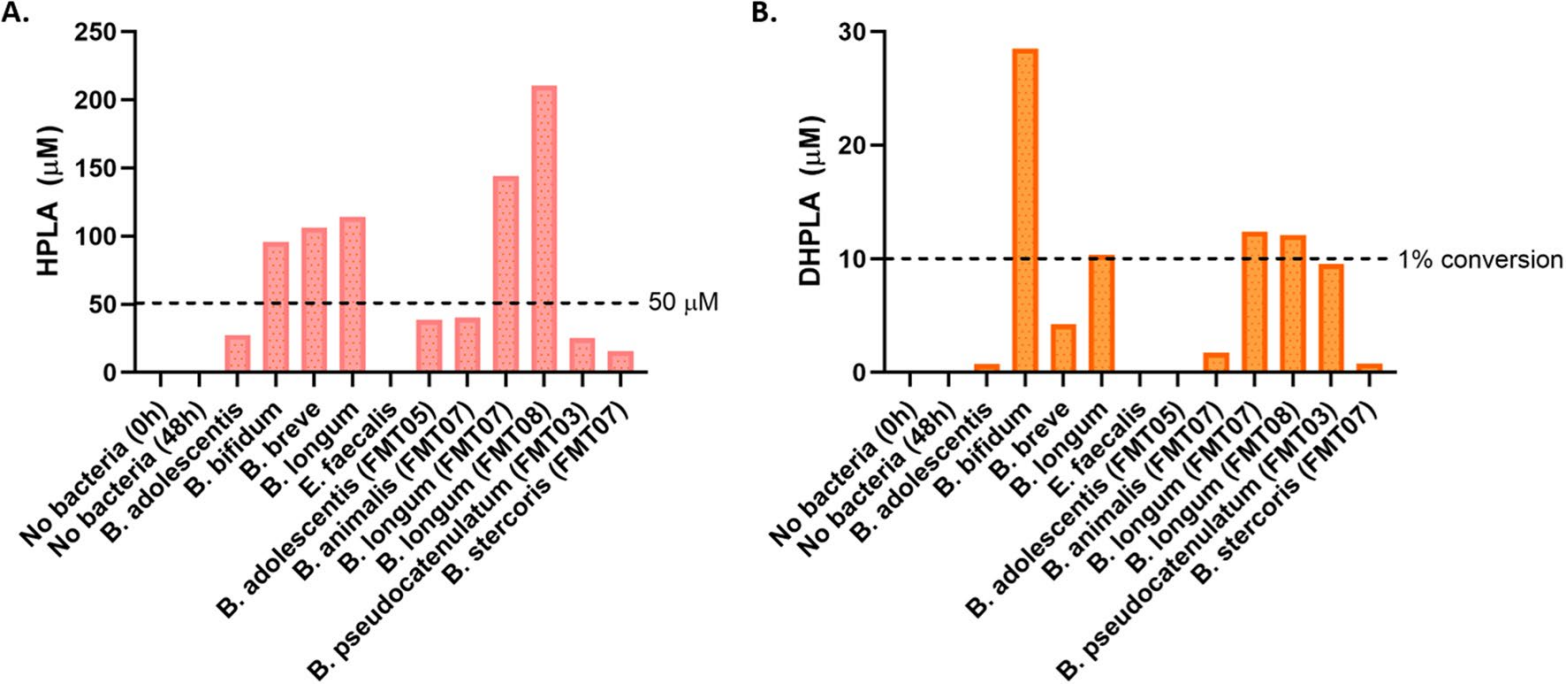
Production of HPLA and DHPLA by *Bifidobacterium*. (**A**) Production of the tyrosine metabolite HPLA by various strains of *Bifidobacterium*. (**B**) Production of the L-DOPA metabolite DHPLA by the same bacteria.

Reference genomes of all utilized *Bifidobacterium* species were searched for annotated lactate dehydrogenase genes using NCBI GenBank, and it was found—as also described by Laursen et al.^8^—that *B. longum*, *B. bifidum*, and *B. breve* contain a lactate dehydrogenase in close proximity to an aromatic amino acid aminotransferase in what may be a potential operon (Supplementary Fig. 2). To test the functionality of these genes, *Bifidobacterium* strains were screened for their ability to produce 4-hydroxyphenyl lactic acid (HPLA) from MRS-C media, which naturally contains tyrosine. It was found that *B. bifidum, B. breve,* and all strains of *B. longum* produced relatively high amounts of HPLA (Fig. 3A), while strains of *B. adolescentis, B. animalis, B. stercoris,* and *E. faecalis* did not. These data match the findings by Laursen et al.^8^.

### Conversion of L-DOPA to the aromatic lactic acid DHPLA

Bacteria were then screened for their ability to convert L-DOPA to the equivalent aromatic lactic acid, 3,4-dihydroxyphenyl lactic acid (DHPLA), from MRS + C medium supplemented with 1 mM L-DOPA. There was high concordance between bacteria able to produce HPLA from tyrosine, and those able to generate DHPLA from L-DOPA (Fig. 3B). However, conversion rates to DHPLA were low, with only four isolates producing > 10 µM DHPLA which represents 1% conversion of the initial 1 mM L-DOPA. Importantly, there was no spontaneous generation of DHPLA in media without bacteria, nor with *E. faecalis* which served as a negative control. Therefore, it was concluded that several *Bifidobacterium* species possess this metabolic capacity, however in a nutrient-rich medium, this reaction appears to exhibit limited activation, and/or L-DOPA is not the preferred substrate.

**Figure 4:**
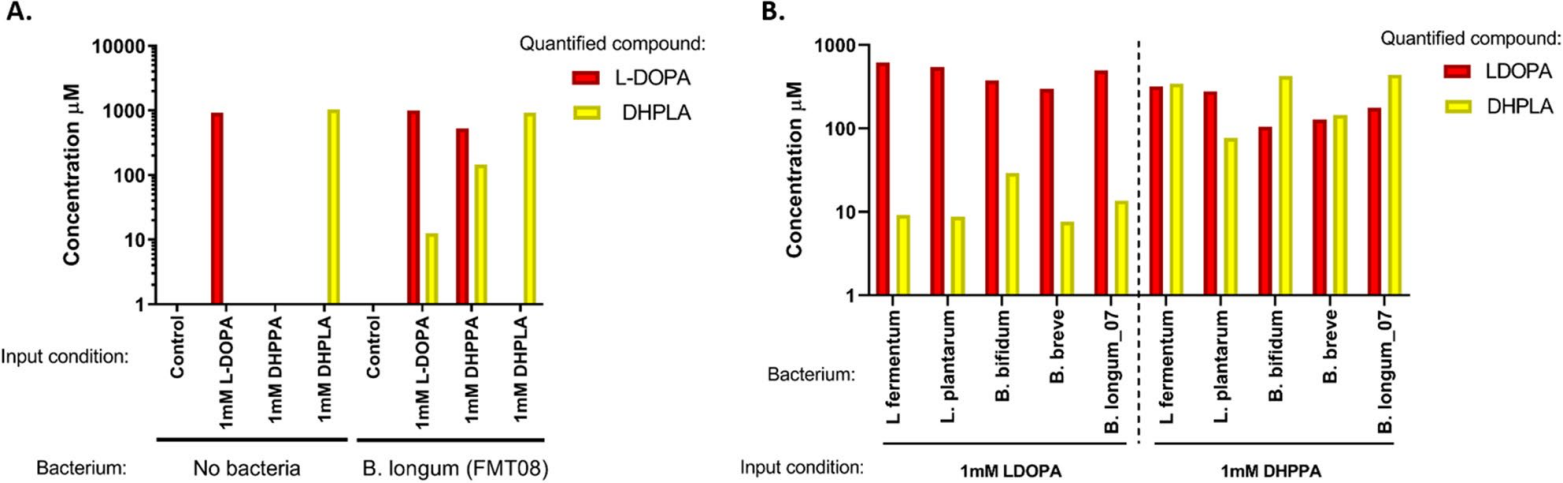
*Bifidobacteria* prefer to re-aminate DHPPA in rich medium. Bacteria were cultured in MRS + C medium with 1 mM of L-DOPA, the intermediate DHPPA, or the end-product DHPLA. (**A**) *B. longum* converted a small amount of L-DOPA to DHPLA. When incubated with DHPPA, it reduced ~ 15% to DHPLA and the remainder was re-aminated back to L-DOPA. When incubated with DHPLA, no further metabolism occurred. (**B**) This experiment was repeated using additional strains with similar results.

To investigate the dynamics of this reaction, *B. longum_08* was incubated with either 1 mM L-DOPA, 1 mM DHPPA, or 1 mM DHPLA; and L-DOPA and DHPLA were quantified after 48 h (Fig. 4A). In controls without bacteria, 100% of the starting compound was recovered, indicating no spontaneous breakdown. With bacteria + L-DOPA, *B. longum* converted ~ 2% into DHPLA, as previously seen. Interestingly, with bacteria + DHPPA, *B. longum* converted ~ 15% to DHPLA, and the remainder was actually converted back to L-DOPA. This indicates that the transamination reaction is skewed in the direction of making L-DOPA (*i.e.* making tyrosine under normal conditions) in rich medium. In the condition with bacteria + DHPLA, 100% DHPLA was recovered and L-DOPA was not detected, revealing that DHPLA is indeed a final metabolic end-product and suggesting that any generation of DHPLA is irreversible. This experiment was repeated with the remaining bacteria of interest, with similar results (Fig. 4B). Bacteria + L-DOPA converted ~ 1–3% of the L-DOPA to DHPLA, and when DHPPA was the starting compound, they variably converted between ~ 10–40% to DHPLA and the remainder back to L-DOPA. All bacteria incubated with DHPLA resulted in 100% DHPLA recovery. In conclusion, this demonstrates that the deamination reaction (L-DOPA → DHPPA) is the rate-limiting step in the conversion of L-DOPA to DHLPA and that it is balanced in the reverse direction in nutrient-rich medium, but that any L-DOPA that is converted to DHPLA is permanently lost as a waste product.

### Enzymatic capability for L-DOPA metabolism

**Figure 5:**
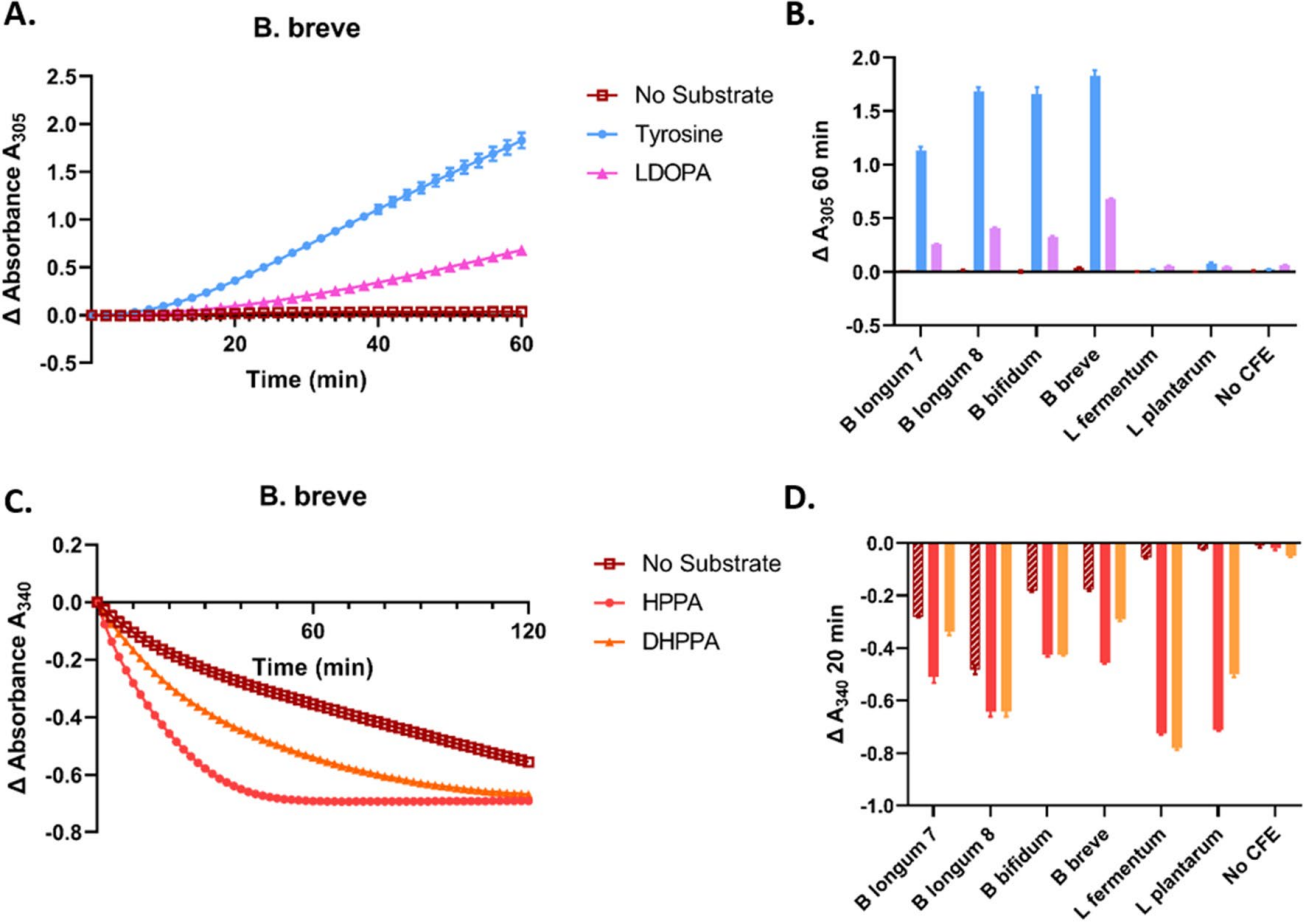
Cell-free extract (CFE) assays demonstrate L-DOPA metabolism potential. (**A**) Representative aminotransferase (AAT) assay with *B. breve.* With no substrate, no amino-group transfer to α-ketoglutarate (detected by an increase in A_305_) occurs. With tyrosine, and L-DOPA at a slower rate, this reaction takes place. A_305_ at time = 0 are normalized to 0. (**B**) Summarized results of the change in OD_305_ after 60 min for all bacteria. (**C**) Representative lactate dehydrogenase (ALDH) assay for *B. breve* in which oxidation of NADH to NAD + causes a decrease in A_340_. Here, significant background activity occurred without any substrate, however all bacteria performed this reaction above background levels as summarized in (**D**). In both assays, no reactions occurred without CFEs.

We next more closely investigated the kinetics of both the deamination (L-DOPA → DHPPA) and reduction (DHPPA → DHPLA) reactions using cell-free-extract (CFE) enzymatic assays. In addition to the *Bifidobacterium* isolates of interest, two environmental *Lactobacilli* (*L. fermentum* and *L. plantarum*) were used due to their known ability to produce high amounts of the tyrosine metabolite HPLA^22–24^. The first assay measured AAT activity, which deaminates tyrosine to HPPA and L-DOPA to DHPPA. The two *Lactobacilli* did not have this capability; however, all four *Bifidobacteria* tested were able to perform this reaction (Fig. 5A,B). In each case, the reaction was faster with tyrosine than with L-DOPA, which is unsurprising as tyrosine is the natural substrate.

The second assay measured ALDH-mediated reduction of HPPA to HPLA and DHPPA to DHPLA. The two *Lactobacilli*, as expected, performed this reaction with both substrates extremely efficiently. The four *Bifidobacteria* also carried out the reaction with both substrates, albeit at a slower rate, and with higher background activity (*i.e.* other components in the CFE were also potential substrates) (Fig. 5C,D). For both enzymatic assays, negative controls without CFEs showed no background activity.

### L-DOPA metabolism in minimal media

To investigate whether the metabolism of L-DOPA by *Bifidobacterium* is upregulated in a nutrient-limited environment, a defined minimal media (BMM) was employed. Of all strains, only *B. breve* was able to grow in this medium without exogenous amino acids and was thus the focus of these experiments. Growth in this media supplemented with 1 mM L-DOPA for 48 h resulted in 126.2 µM DHPLA production as measured by LC–MS/MS, representing ~ 12% conversion, significantly higher than the ~ 1% observed in rich MRS + C medium. No DHPLA was produced in BMM without L-DOPA, ruling out spontaneous production of this molecule.

**Figure 6:**
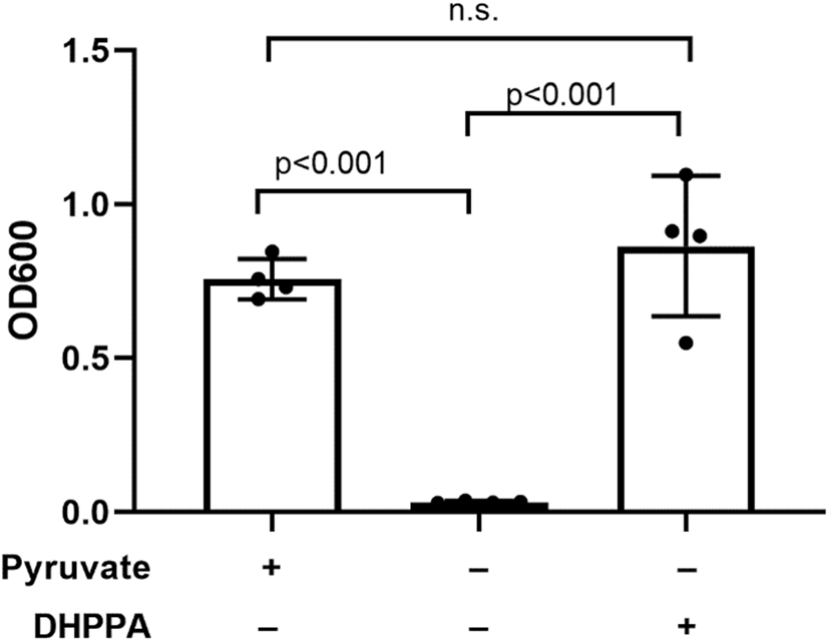
Minimal medium replacement experiment. In minimal medium, pyruvate is essential for *B. breve* growth. The L-DOPA metabolite DHPPA is able to replace pyruvate and restore growth. Cultures were grown for 48 h.

According to the proposed pathway of L-DOPA metabolism, the final step—conversion of DHPPA to the metabolic end-product DHPLA—is a redox reaction used by the bacteria to regenerate NAD + from NADH. In the minimal medium, this anaerobic homolactic fermentation reaction occurs via conversion of pyruvate to lactic acid. Removal of pyruvate from BMM resulted in complete inhibition of *B. breve* growth. When an equimolar amount of DHPPA was added to the media, growth was completely restored (Fig. 6), confirming that *B. breve* can use this L-DOPA metabolite for the essential function of NAD + regeneration.

## Discussion

To provide dependable symptom relief, it is critical that L-DOPA reaches the brain intact. It has recently become clear that in addition to host peripheral metabolism, metabolism by gut bacteria may also be a concern^6,7^. This study describes a novel pathway of L-DOPA metabolism by certain *Bifidobacterium* species employing existing tyrosine metabolism genes. In PD patients, we observed a negative correlation between *Bifidobacterium* and serum tyrosine. This is similar to the pattern seen in fecal samples from breastfed infants^8^, revealing strong evidence for tyrosine metabolism by these bacteria in vivo*.* A recent study also observed higher *Bifidobacterium* and lower concentrations of aromatic amino acids in PD fecal samples^25^.

*Bifidobacteria* were not able to decarboxylate L-DOPA to dopamine, a pathway recently observed in *Enterococcus* and *Eggerthella* species^6,7^; therefore, we searched for alternative pathways to explain our clinical observations. Non-commensals—including pathogens and environmental bacteria—frequently encode aromatic amino acid aminotransferases^26–28^ that are possibly able to deaminate L-DOPA to DHPPA, and lactate dehydrogenases^8,29,30^ that could reduce DHPPA to DHPLA. Indeed, a recent study found that the soil bacterium *Clostridium sporogenes* can efficiently deaminate L-DOPA to DHPPA using an aminotransferase^31^. Our study describes the first observation of the complete metabolic pathway from L-DOPA to the aromatic lactic acid DHPLA among common gut commensals.

Compared to the ability of *Enterococcus* to metabolize L-DOPA, *Bifidobacteria* do so much less efficiently; *Enterococcus* has been shown to metabolize 100% of L-DOPA in culture to dopamine within 24h^6,7^ (which we also observed), whereas strains of *Bifidobacteria* converted 1–3% to DHLPA in 48 h in rich media, and approximately 12% in nutrient-limited media. Importantly, however, the reduction of DHPPA to DHPLA appears to be irreversible, with DHPLA being a final end-product analogous to lactic acid generation by these bacteria during homolactic fermentation^32^. Therefore, any DHPLA generated becomes permanently unavailable for reconversion to L-DOPA. In minimal medium, which likely more closely resembles the nutrient-deprived conditions in the colon, substantially more L-DOPA is converted into the final lactic acid end-product by these bacteria.

The strong association we observed between higher *Bifidobacterium* abundance and entacapone use has been previously noted in other studies^33–35^. As a catechol-O-methyltransferase inhibitor, entacapone serves to prolong the half-life of L-DOPA in the body by preventing its degradation by COMT. This would likely increase the fraction of non-metabolized L-DOPA that reaches the colon, where it would become available for microbiota metabolism, thus potentially explaining the positive association between *Bifidobacterium* and entacapone. However, entacapone use has been shown to affect the abundance of other gut bacterial genera as well^33,35^, therefore it may have larger scale effects on the microbiome or on the overall gut environment that are not yet fully understood.

The observed positive correlation between *Bifidobacterium* abundance and L-DOPA dose in PD patients has two possible interpretations. The first is that patients with higher levels of *Bifidobacterium* require higher medication dose because their gut bacteria are metabolizing a significant portion and making it clinically unavailable. The second is that in patients with naturally higher medication doses (for clinical reasons), a growth advantage is conferred to *Bifidobacterium* as they are able to use residual L-DOPA for metabolic purposes. In light of the fairly modest metabolism of this drug, and the fact that the highest microbiota biomass and metabolic activity is in the colon^32^ whereas L-DOPA is absorbed in the upper small intestine^1,3^, the second interpretation is more plausible. Approximately 10% of orally ingested L-DOPA is never absorbed into systemic circulation^31,36,37^, leaving ample substrate for bacterial metabolism in the lower GI tract. Thus, the administration of L-DOPA may provide a fitness advantage to microorganisms harboring this low-affinity transformation pathway. Interestingly, although many human cohort studies report higher levels of *Bifidobacterium* among PD patients, one large study of medication-naïve patients^38^ and another of (also L-DOPA-naïve) individuals with idiopathic rapid eye movement sleep behavior disorder^39^—a condition with extremely high conversion to PD—both observed no differences in *Bifidobacterium* abundance between patients and controls. This perhaps suggests that it is indeed the initiation of L-DOPA administration that drives a subsequent increase in *Bifidobacterium.* This is further supported by the longer disease duration, worse disease severity, and increased entacapone use among PD patients with higher *Bifidobacterium* in our cohort; in other words, it is more likely that patients’ disease advancement and the associated increase in L-DOPA dose (and use of L-DOPA-stabilizing drugs) drives subsequent *Bifidobacterium* flourishing, rather than higher *Bifidobacterium* abundance driving increased dose requirements. This is also borne out by the fact that most microbial metabolism (in the colon) is downstream of the site of clinically-relevant L-DOPA absorption (in the upper small intestine). Large studies specifically investigating changes in microbial composition pre- and post-L-DOPA initiation in newly diagnosed PD patients will further inform this hypothesis.

*Lactobacillus* is another genus that employs homolactic fermentation, and interestingly, this genus has also been reported to be higher in abundance in PD^10,11^. In our cohort, we observed very little of this genus overall (both in patients and controls)^12^, and during the isolation of *Bifidobacterium* strains, no *Lactobacilli* were found despite the fact that they grow well on MRS medium. Therefore, due to the lack of supporting clinical data and isolates, whether this pathway also exists in any commensal gut *Lactobacilli* was not explored; however, it is an intriguing possibility.

This study has several limitations. The human cohort data is associative, and no causality can be ascribed—indeed, we offer two contrasting explanations for the positive correlation between *Bifidobacterium* abundance and L-DOPA dose among PD patients. Our microbiota data was based on 16S analysis which did not discriminate *Bifidobacteria* to the species level, therefore our results group the entire genus, although it is clear that only certain species can perform this reaction. The in vitro studies are limited by the fact that they are single-organism in nature. In reality, the microbiota is a complex community which involves competition, cross-feeding, and environmental conditions that cannot be replicated in a culture tube.

We urge caution against over-interpreting these results. As mentioned, the efficiency of this L-DOPA metabolism reaction in *Bifidobacterium* is quite low, reaching only ~ 1% in complete medium and only ~ 12% in minimal medium after 48 h. Indeed, in nutrient-rich medium, the aminotransferase reaction is actually balanced in the direction of regenerating L-DOPA from the intermediate pyruvic acid, implying that in certain contexts *Bifidobacteria* could actually protect the integrity of L-DOPA against bacteria that efficiently deaminate (but not reduce) it. *Bifidobacterium*-containing probiotics have shown benefits in many aspects that may be relevant to PD, including improving mood and gastrointestinal function^40–43^. Therefore, if PD patients report positive experiences from probiotic use, we suggest that the benefits likely outweigh the possible low-level metabolism of residual L-DOPA by these species and we do not suggest discontinuing probiotic administration based on the results of this study.

As a final note, beyond direct metabolism by gut bacteria, other factors related to gut health also impact the pharmaceutical efficacy of L-DOPA. Gastroparesis, slow colonic motility, and constipation can all negatively impact L-DOPA absorption and lead to unpredictable “OFF” periods^5^. Therefore, any interventions, microbial or otherwise, that restore normal gut health and function could have the added benefit of aiding with medication management and symptom relief in this population.

### Supplementary Information

Supplementary Information.

## Data Availability

Raw 16S sequencing data and anonymized patient demographic information, including medication dose, is available at NCBI SRA accession PRJNA594156.
