# Supplementary material for: A novel pathway of levodopa metabolism by commensal Bifidobacteria
Source: Sci Rep. 2023 Nov 6;13:19155. doi: 10.1038/s41598-023-45953-z (PMC10628163; doi:10.1038/s41598-023-45953-z)
Supplement: Supplementary file 1 — Supplementary Information. [file 41598_2023_45953_MOESM1_ESM.docx]

**
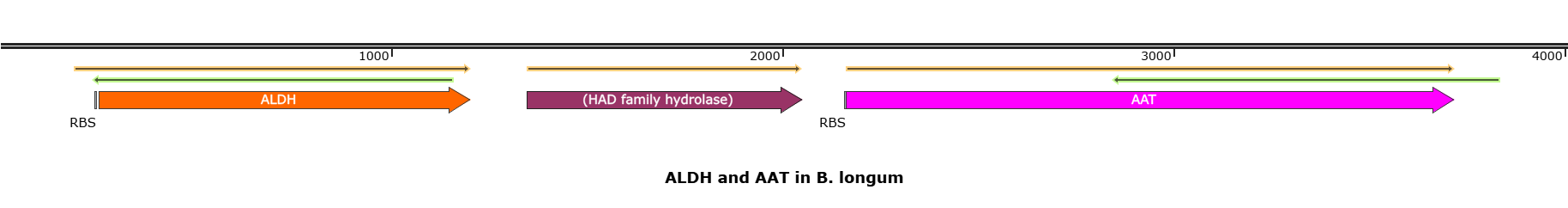
Supplementary Figure 1. Potential tyrosine metabolism operon in *Bifidobacterium*.** A lactate dehydrogenase (ALDH) and aromatic amino acid aminotransferase (AAT) gene are found in close proximity, in a possible operon, in *B. breve, B. bifidum,* and *B. longum.*

**
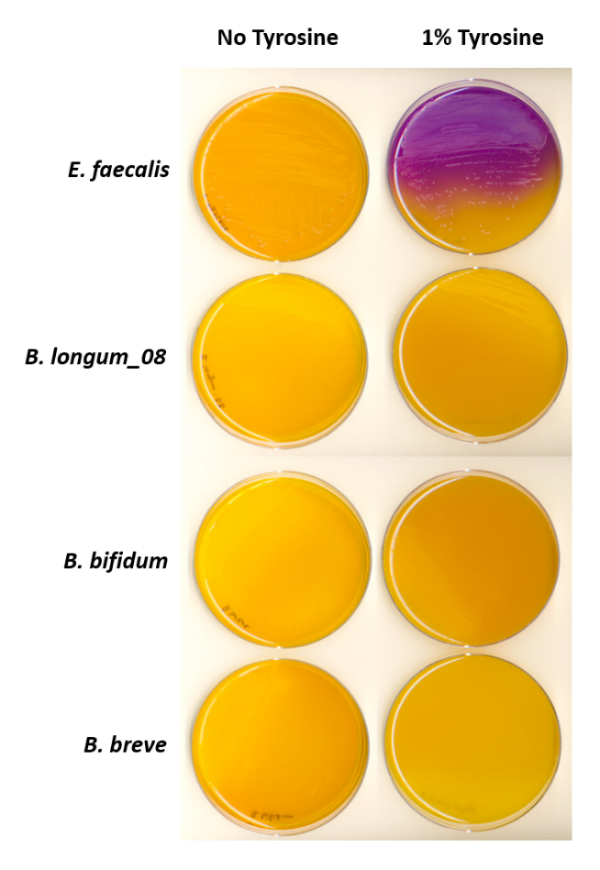
**

**Supplementary Figure 2. No observed tyrosine decarboxylation activity in *Bifidobacterium*.** A pH-shift-associated colour change from yellow to purple indicates tyrosine decarboxylation to tyramine in tyrosine-containing plates. The positive control *Enterococcus faecalis* readily decarboxylated tyrosine, however none of the assayed *Bifidobacterium* strains had this ability.

**Supplementary Table 1. Decarboxylase medium formulation.**

| **Ingredient** | **Final % w/v** |
| --- | --- |
| Tryptone | 0.5 |
| Yeast extract | 0.5 |
| Beef extract | 0.5 |
| Sodium chloride | 0.25 |
| Glucose | 0.05 |
| Tween 80 | 0.1 |
| MgSO_4_ heptahydrate | 0.04 |
| MnSO_4_ hydrate | 0.0056 |
| Fe_2_(SO_4_)_3_ hydrate | 0.005 |
| Ammonium citrate | 0.2 |
| Thiamine hydrochloride | 0.0012 |
| K_2_PO_4_ | 0.2 |
| NaHCO_3_ | 0.008 |
| Pyridoxal-5-phosphate | 0.005 |
| Bromocresol purple | 0.006 |
| Bacto-Agar | 1.7 |
| Tyrosine * | 1 |

* Control media has no tyrosine

- Make the above formulation and adjust pH to 5.25
- Autoclave for 10 min
- When cooled to ~55°C, add 1X ATCC Vitamin Mix then pour plates.

**Supplementary Table 2. L-DOPA experiment LC-MS/MS parameters**

| Molecule | Molecular formula | Exact mass [M] | Precursor ion | Fragment ion | Dwell | Fragmentor Energy (V) | Collision Energy (V) | Cell Accelerator (V) | Polarity |
| --- | --- | --- | --- | --- | --- | --- | --- | --- | --- |
| L-DOPA-d_3_ (IS) | C_9_H_8_D_3_N_1_O_4_ | 200.0876 | 201.0949 | 154.09 | 100 | 70 | 10 | 7 | + |
| Dopamine | C_8_H_11_NO_2_ | 153.0789 | 154.0862 | 137.06 | 200 | 60 | 5 | 7 | + |
| LDOPA | C_9_H_11_N_1_O_4_ | 197.0688 | 198.0760 | 152.07 | 100 | 100 | 8 | 7 | + |
| DHPLA | C_9_H_10_O_5_ | 198.0528 | 197.0455 | 135.02 | 100 | 70 | 10 | 7 | - |
| HPLA | C_9_H_10_O_4_ | 182.0579 | 181.0506 | 163.04 | 100 | 80 | 8 | 7 | - |

Optimized mass spectrometer settings for analytes evaluated in LC-MS/MS.

**Supplementary Table 3. *Bifidobacterium*** **minimal medium (BMM) formulation.**

| **Ingredient** | **[Final]** | **Notes** |
| --- | --- | --- |
| D-Lactose | 3.50% | (1) |
| Sodium acetate | 2.50% | (1) |
| Ammonium acetate | 0.20% | (1) |
| Dipotassium phosphate | 0.25% | (1) |
| D-Pantethine | 50 µg/mL | (2) |
| Sodium pyruvate | 0.25% | (2) |
| L-Cysteine-HCl | 0.10% | (2) |
| MgCl_2_ | 0.5 mM | (3) |
| MnCl_2_ | 0.5 µM | (3) |
| CaCl_2_ | 50 µM | (3) |
| Hemin | 5 µg/mL | (4) |
| ATCC Vitamin Mix | 1X | (5) |

(1) Combined and dissolved in H_2_O as a 4X stock

(2) Dissolved in H_2_O as a 100X stock (each ingredient separately)

(3) Combined and dissolved in 10% L-Cysteine-HCl in H2O as a 100X stock

(4) Dissolved in H_2_O with concentrated NaOH added dropwise until soluble; sterile filtered as a 1000X stock

(5) Purchased as a 100X stock

All stocks were sterile filtered. To make (*e.g.*) 1L media: Combine 745 mL sterile H_2_O, 250 mL of (1), and the remaining stocks (2-4) diluted to 1X.
